# The effect of implant‐abutment connections on peri‐implant bone levels around single implants in the aesthetic zone: A systematic review and a meta‐analysis

**DOI:** 10.1002/cre2.471

**Published:** 2021-08-21

**Authors:** Caroliene M. Meijndert, Gerry M. Raghoebar, Arjan Vissink, Konstantina Delli, Henny J. A. Meijer

**Affiliations:** ^1^ Department of Oral and Maxillofacial Surgery University of Groningen, University Medical Centre Groningen Groningen The Netherlands; ^2^ Department of Implant Dentistry, Dental School University of Groningen, University Medical Center Groningen Groningen The Netherlands

**Keywords:** aesthetic region, bone level, implant‐abutment connection, systematic review

## Abstract

**Objective:**

To systematically review literature about the effect of different implant‐abutment interface designs on peri‐implant bone level changes, implant loss and mid‐buccal mucosa changes around single implants in the anterior maxilla. Reviewing three connection configurations: Platform switched conical (PS‐conical); Platform switched parallel (PS‐parallel); Platform matched parallel (PM‐parallel).

**Methods:**

A detailed search was carried out in Pubmed, EMBASE, Cochrane, Scopus, Open Gray and African journals Online (until December 1, 2020) and was restricted to clinical prospective studies of at least 1 year and with at least 10 human participants. A meta regression analysis was carried out primarily on the pooled peri‐implant bone level changes followed by implant loss and mid‐buccal mucosa level change. Risk of bias was assessed with RoB 2.0 and ROBINS‐I. The manuscript complied with the PRISMA guidelines and was registered in the PROSPERO database (ID: 225092).

**Results:**

A total of 5513 hits gave 44 eligible articles for the analyses. Bone level change did not differ significantly between the two platform switched connections; their bone loss scores were significantly lower than PM‐connection. The PS‐conical connections have significantly lower implant losses than the PM connection. Mid‐buccal mucosa level change was comparable between the three connection configurations. Moderate to high risk of bias was detected in the included studies.

**Conclusions:**

The performance of PS‐conical and PS‐parallel connection configurations both favored bone loss scores compared to the PM‐parallel connection configuration. All three demonstrated mid‐buccal mucosa changes that were small and did not differ significantly amongst the groups.

## 1 INTRODUCTION

The range of implants used in restorative dentistry has become increasingly sophisticated and, simultaneously, more complex (Buser et al., 2017). The variation has become so large, that it is difficult for clinicians to choose between the various available components (Shafie & White, 2014). One of these components is the implant‐abutment connection configuration. A clear distinction can be made between external and internal implant‐abutment connections. External connections are hardly used anymore because of the susceptibility to complications (Gracis et al., 2012; Koo et al., 2012; Steinebrunner et al., 2005). Internal connections have a number of geometric variations (Koutouzis, 2019). For example, the internal implant geometry is parallel‐walled or conical/tapered. The parallel‐walled connection types are often equipped with various indexing/anti‐rotational features like an octagon or a hexagon, and exist with a platform switch or a platform match. A conical/tapered connection type implicates a cone‐in‐cone or has a conical portion in the inner cervical part, with or without indexing features in the apical part (Shafie & White, 2014).

All the previous mentioned variations were developed in an effort to reduce mechanical failure (Ceruso et al., 2017) and to minimize crestal bone resorption (Koutouzis, 2019). It is presumed that the long‐term survival and success of implant treatment can be affected by peri‐implant crestal bone resorption (Schwartz‐Arad et al., 2005). This bone resorption can affect the stability of the mucosa, and may therefore affect the aesthetic outcome, which is particularly interesting in the aesthetic zone (Belser et al., 2009; Fürhauser et al., 2005; Jemt, 1997). Eliminating, or at least reducing the amount of bacterial leakage, could have a positive influence on peri‐implant tissue stability, and thus on the aesthetics. It has been suggested that an internal implant‐abutment connection with a conical configuration is the most stable connection, with less bacterial leakage, than the other configurations (Zipprich et al., 2018).

In 2018, Caricasulo et al. (2018) performed a systematic review, with a meta‐analysis, of the difference between conical, internal and external connection configurations. They concluded that significantly less bone loss occurs with conical and internal connections compared to external connections. Although they did not distinguish between platform switching and platform matching, they concluded after performing an additional analysis that a platform switch might be of more importance in preserving peri‐implant bone levels than the connection configuration itself. The finding that platform switching might have a positive effect on preserving peri‐implant bone levels was also mentioned by Hsu et al. (2017). However, Caricasulo et al. (2018) and Hsu et al. (2017) did not focus specifically on the aesthetic region or on a possible effect on the aesthetic outcome.

Vetromilla et al. (2019) did perform a systematic review on implant abutment connections in the aesthetic region. They concluded from an aesthetic scoring index by professionals, being the Pink Esthetic Score and White Esthetic Score (PES/WES; Belser et al., 2009) that the internal hexagon performs better aesthetically, but were not able to quantify their observations. Additionally, they did not distinguish between platform‐switched and platform‐matched connections, but this might be a noteworthy nuance when considering the Caricasulo et al. (2018) outcome.

Since the aesthetic outcome of a restoration in the aesthetic region is so important, it would be interesting to know if the implant abutment connection can contribute to achieving the best possible results. So far, to the best of our knowledge, a systematic review with a meta‐analysis of the effect of solely internal connections in the aesthetic region, distinguishing between platform switching and matching, is not available. Therefore, the aim of our systematic review was to determine whether the type of implant connection configuration, that is, platform switched conical connections (PS‐conical), platform switched parallel connections (PS‐parallel) or platform matched parallel connections (PM‐parallel), has a significant impact on peri‐implant bone changes, whether an implant‐abutment connection influences implant survival, and whether the stability of the mid‐buccal mucosa level, as a factor determining the aesthetic outcome, is influenced by the connection configuration.

## 2 METHODS

### 2.1 Research protocol

This systematic review was conducted following the Cochrane Handbook for systematic reviews and was reported according to the PRISMA Statement guidelines 2009 (Moher et al., 2009). The protocol of this systematic review was entered under the PROSPERO registration number: ID: 225092. The research question was formulated by means of a PICO:

P—solitary implants in the maxillary aesthetic region with titanium implants.

I—conical implant‐abutment connections.

C—parallel walled implant‐abutment connections with and without a platform switch.

O—peri‐implant bone level change.

The primary outcome is peri‐implant bone level change and the secondary outcome are implant loss and mid‐buccal mucosa level change.

### 2.2 Information sources

We conducted a literature research of the following databases: PubMed, Cochrane Library EMBASE Scopus, Open Grey and African journals Online. According to the syntax rules of each database, key words and their combinations were used to identify studies published until December 2020. No time restrictions were applied (Table 1).

**TABLE 1:** Search strings

Pubmed (1673 hits)	
(“Dental Implants, Single‐Tooth” [Mesh] OR (implant*[tiab] AND (single[tiab] OR solitary[tiab]))) AND (“Maxilla” [Mesh] OR “Esthetics, Dental” [Mesh:NoExp] OR esthetic*[tiab] OR aesthetic*[tiab] OR anterior[tiab] OR maxilla*[tiab] OR incisor*[tiab] OR front*[tiab]) AND (“Alveolar Bone Loss” [Mesh] OR bone[tiab]) AND (“Clinical Trial” [Publication Type] OR “Cohort Studies” [Mesh] OR “Case Reports” [Publication Type] OR “Observational Study” [Publication Type] OR “Treatment Outcome” [Mesh:NoExp] OR “Comparative Study” [Publication Type] OR random*[tiab] OR trial[ti] OR outcome*[tiab] OR cohort[tiab] OR follow‐up[tiab] OR followup[tiab] OR prospectiv*[tiab] OR longitudinal*[tiab] OR case ser*[tiab])	
Embase (1539 hits)	
(“single tooth implant”/exp OR (implant* AND (single OR solitary)):ab,ti) AND (“maxilla”/exp OR “esthetics”/exp OR (esthetic* OR aesthetic* OR anterior OR maxilla* OR incisor* OR front*):ab,ti) AND (“alveolar bone loss”/exp OR bone:ab,ti) AND (“clinical study”/exp OR “observational study”/exp OR “cohort analysis”/exp OR “comparative study”/exp OR “treatment outcome”/de OR trial:ti OR (random* OR outcome* OR cohort OR “follow‐up” OR followup OR prospectiv* OR longitudinal* OR “case ser*”):ab,ti)	
Cochrane (527 hits)	
(implant* AND (single OR solitary)) AND (esthetic* OR aesthetic* OR anterior OR maxilla* OR incisor* OR front*) AND (bone)	
Scopus (1626 hits)	
(TITLE‐ABS‐KEY (implant* AND ( single OR solitary))) AND (TITLE‐ABS‐KEY (dental OR tooth OR teeth OR crown*)) AND (TITLE‐ABS‐KEY (esthetic* OR aesthetic* OR anterior OR maxilla* OR incisor* OR front*)) AND (TITLE‐ABS‐KEY (bone)) AND (TITLE‐ABS‐KEY (“clinical trial*” OR prospectiv* OR cohort* OR “case report*” OR “case stud*” OR observational* OR “follow‐up” OR followup OR random* OR outcome* OR longitudinal* OR “case ser*” OR “clinical stud*” OR “controlled stud*”))	
Open Grey (24 hits)	
Single implant	
African Journal *Online* (145 hits)	
Dental AND implant AND single AND maxilla AND bone	

The following study criteria were handled:

Inclusion criteria:

- Human subjects included in the studies should be ≥18 years of age.
- Titanium, bone level implants.
- Implants supporting single crowns placed in the anterior region of the maxilla (second premolar to second premolar).
- Only healed sites (at least 3 months healing time after extraction) and either or not a bone augmentation procedure has been performed in an earlier session.
- Follow‐up of at least 1 year after implant placement.
- Detailed information on bone level changes measured on peri‐apical radiographs.
- Randomized clinical trials or prospective clinical studies with a minimum sample of 10 participants per study group.

Exclusion criteria:

- External implant‐abutment connections.
- No details of the implant type.
- Did not report bone level changes.
- Bone level measurements obtained with cone beam computer tomography (CBCT) or orthopantomograms.
- Animal studies, in vitro studies, retrospective studies, reviews.

The impact of implant‐abutment connection already starts at the time of connection and a possible reaction of surrounding hard and soft tissues to the amount of bacterial leakage can be found within the first year of functioning. Therefore, a minimum follow‐up time of 1‐year is considered as meaningful.

Mean marginal bone change was defined as the mean value of the change in marginal bone level at the mesial and distal side of the implant, measured along the implant axis and calculated as the difference in bone level between start of the functional period and the follow‐up evaluation.

Studies with immediate implant placement were not included. With immediate implant placement the first bone‐implant contact at start of the evaluation period is not in the area of the neck of the implant. It takes a certain period of time to heal for the remaining socket around the implant. This healing period could possibly interfere with possible bone level changes due to the implant‐abutment connection.

Both screw‐retained and cement‐retained single crowns were included. There is no evidence in the literature that one type is performing better with respect to marginal bone level changes.

### 2.3 Study selection

Two reviewers (C.M.M., H.J.A.M.) independently screened the results from the electronic searches, according to the inclusion and exclusion criteria, in two rounds. Articles were first screened by title and abstract. In case of disagreement or doubt, the study was moved to the next round (full text assessment). The Cohen's *κ* and percentage of agreement were calculated to determine the measure of agreement between the two reviewers. Any disagreement regarding the inclusion was resolved by a consensus discussion. In case of persistent disagreement, an external independent reviewer (G.M.R.) with experience in implant dentistry could be consulted.

### 2.4 Quality assessment

Methodological quality and risk of bias were assessed using the Cochrane risk of bias tool (RoB 2.0; Sterne et al., 2019) for randomized controlled trials and the ROBINS‐1 tool (Sterne et al., 2016) for prospective clinical non‐randomized trials by the same two reviewers (C.M.M., H.J.A.M.), independently. Any disagreement was resolved by consensus between the reviewers.

### 2.5 Data extraction

Following a pre‐specified form, the following data were extracted cooperatively by C.M.M. and H.J.A.M.: authors, year of publication, study design, follow‐up time, type of implant, type of implant‐abutment connection, number of implants, number of implant failures, bone level changes and, if available, additional data on the mid‐buccal mucosa level changes. It was decided to group the studies according to the properties of the internal configuration. When an implant connection was fully or partially conical/tapered along the inner wall of the implant body and the corresponding portion of the abutment, the implant was classified as conical and placed in the “platform switched conical” group (PS‐conical). Any connection where the inner portion of the implant and abutment was parallel‐walled were classified based on the presence or absence of a platform switch and were placed accordingly into either the “platform switch parallel” (PS‐parallel) or “platform matched parallel” group (PM‐parallel).

### 2.6 Statistical analysis

Inter‐observer agreement was calculated with IBM SPSS Statistics 20 (SPSS, Chicago, IL). Publication bias was assessed by plotting the log odds ratio against its standard error. Pooling of implant survival, bone loss and mid buccal mucosa changes was performed by using the software Comprehensive Meta‐Analysis, Version 3.3.070 (CMA, Biostat, Englewood, NJ). A random effects model was used on the pooled outcomes. To analyze sources of heterogeneity between studies, a meta‐regression analysis (random effects model) was performed on the connection types, that is, PS‐conical, PS‐parallel and PM‐parallel.

## 3 RESULTS

### 3.1 Study identification and selection

**FIGURE 1:**
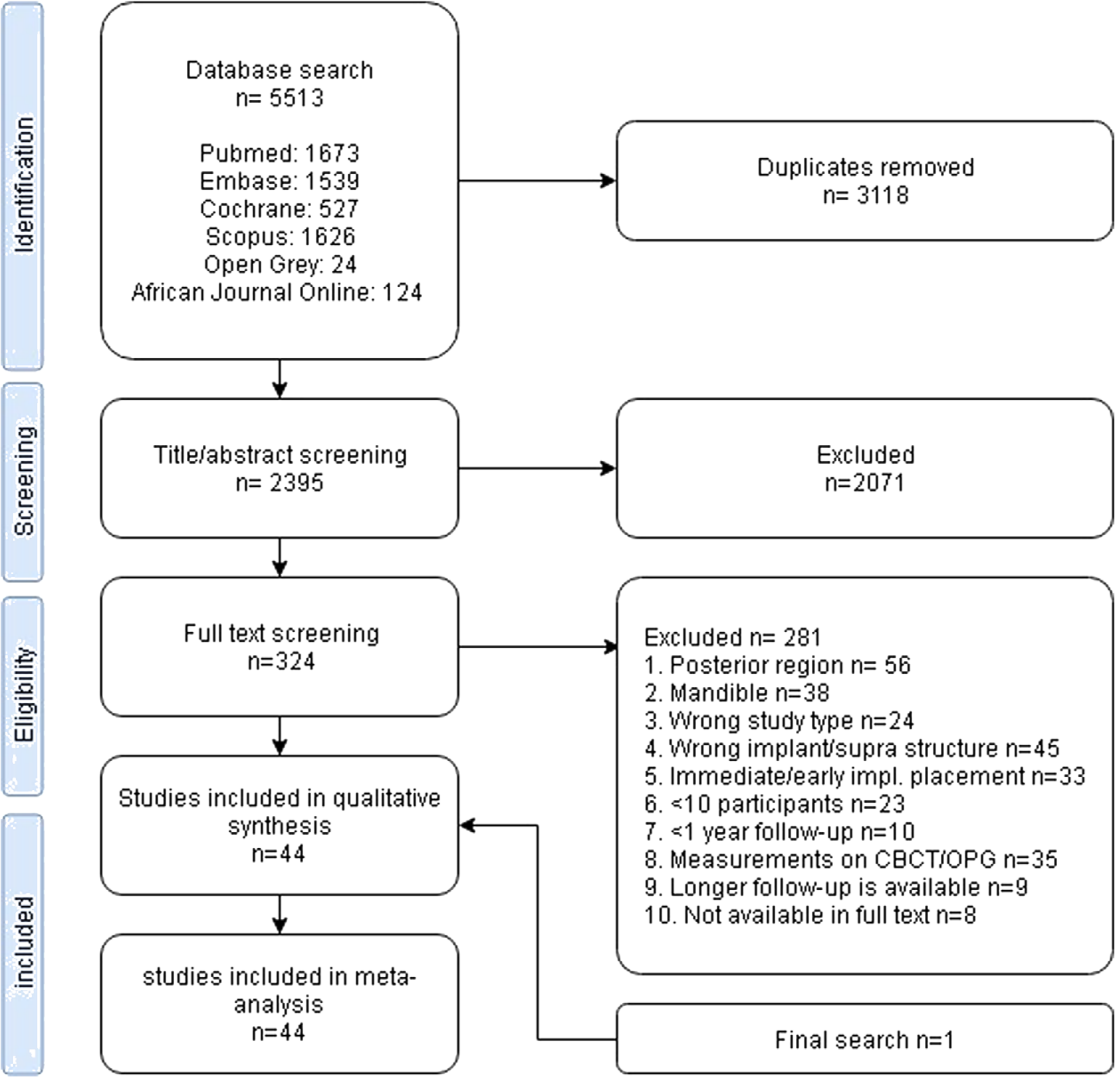
Study identification and selection process. A total of 43 studies were included following the main screening process (up to July 1, 2020). The updated search resulted on December 1, 2020 resulted in one additional study

The study selection procedure is summarized in Figure 1. A total of 5513 publications was identified after the electronic and hand search, up to the July 1, 2020. After discarding the duplicates from the output, a total of 2395 publications underwent title abstract selection whereupon 2071 did not meet the inclusion criteria. A total of 324 articles remained for full‐text analysis and, of these, 281 did not meet the in‐ and exclusion criteria, or they were a follow‐up of an earlier study. A last update was done on the December 1, 2020 and, of the 111 new results, 1 was suitable for analysis. A total of 44 publications was finally included for data extraction. There was substantial agreement between the two reviewers' judgments, *κ* = 0.76, (93.9% agreement) at title/abstract selection. At full text selection, this agreement was also substantial, *κ* = 0.75, (91.4% agreement). There was no need to consult the third reviewer in any of the study selection phases.

### 3.2 Description of the selected studies

The search results yielded 24 non‐randomized clinical trials (nRCTs) and 20 randomized clinical trials (RCTs). The studies' median follow‐up was 1 [1;5] year (ranging from 1 to 10 years). Detailed data from the included studies is listed in Table 2. If an article reported data on two groups, but the same connection type was used, the groups were pooled. When an article reported data from two groups with different connection types, the groups were viewed separately. Consequently, a total of 31 groups (1105 implants) had PS‐conical connections, 5 groups (124 implants) had a PS‐parallel connection, and 12 groups (356 implants) had a PM‐parallel connection.

**TABLE 2:** Characteristics and outcomes of the included studies

	Reference	Publication year	Follow‐up time (years)	Study type	Implant type	No of implants	Annual implant loss (%)	Annual bone level change in mm (mean ± *SD*)	Annual mid‐buccal level change in mm. (mean ± *SD*)
PS‐conical	Kemppainen et al. (1997)	1997	1	RCT	Astra Tech ST	35	2	−0.14 ± 0.10	nr
	Palmer et al. (2000)	2000	5	Prosp. study	Astra Tech ST	14	0	0.02 ± 0.10	nr
	Cooper et al. (2001)	2001	1	Prosp. study	Astra Tech ST	53	4	−0.40 ± 0.51	0.34 ± 0.94
	Norton (2004)	2004	1	Prosp. study	Astra Tech ST	11	4	−0.55 ± 0.49	nr
	Cooper et al. (2007)	2007	3	Prosp. study	Astra Tech ST	43	2	−0.14 ± 0.32	0.17 ± 0.47
	Gotfredsen (2012)	2012	10	Prosp. study	Astra Tech ST	10	0	−0.09 ± 0.04	nr
	Vanlioglu et al. (2012)	2012	5	Prosp. study	Astra Tech OsseoSpeed	10	0	−0.05 ± 0.01	nr
	Bashutski et al. (2013)	2013	1	RCT	Astra Tech OsseoSpeed	24	8	−0.53 ± 0.51	nr
	Grandi et al. (2013)	2013	1	Prosp. study	JD Evolution	24	4	−0.60 ± 0.51	nr
	Berberi et al. (2014)	2014	5	Prosp. study	Astra Tech OsseoSpeed	20	0	−0.04 ± 0.03	nr
	Cooper et al. (2014)	2014	5	Prosp. study	Astra Tech OsseoSpeed	49	0	0.02 ± 0.26	0.08 ± 0.21
	Vanlioglu et al. (2014)	2014	1	Prosp. study	Straumann Bone Level implant	55	0	−0.10 ± 0.08	nr
	Cosyn et al. (2015)	2015	1	Prosp. study	Nobel Active	50	0	−0.48 ± 0.46	0.06 ± 0.60
	Esposito et al. (2015)	2015	1	RCT	Mega Gen, EZ Plus	46	0	−0.27 ± 0.14	nr
	Raes et al. (2015)	2015	2	Prosp. study	Astra Tech OsseoSpeed	85	2	0.02 ± 0.45	0.10 ± 0.45
	Hsu et al. (2016)	2016	1	RCT	SuperLine Dentium	13	0	−0.21 ± 0.56	−0.38 ± 0.49
	Slagter et al. (2016)	2016	1	RCT	Nobel Active	20	0	−0.65 ± 0.46	nr
	Yildiz et al. (2016)	2016	1	Prosp. study	Straumann Bone Level implant	29	7	0.08 ± 0.82	nr
	Allen et al. (2017)	2017	2	Prosp. study	Straumann Bone Level implant	20	0	−0.41 ± 0.26	nr
	De Bruyckere et al. (2018)	2018	1	RCT	Nobel Active	42	0	−0.60 ± 0.69	−0.18 ± 0.33
	Eghbali et al. (2018)	2018	5	Prosp. study	Nobel Active	32	0	−0.09 ± 0.08	−0.02 ± 0.07
	Jonker et al. (2018)	2018	1	RCT	Straumann Bone Level implant	43	2	−0.42 ± 0.55	nr
	Raes et al. (2018)	2018	8	Prosp. study	Astra Tech OsseoSpeed	18	0	−0.06 ± 0.24	nr
	Zuiderveld et al. (2018)	2018	1	RCT	Nobel Replace CC	40	0	−0.02 ± 0.11	−0.11 ± 1.19
	Cooper et al. (2019)	2019	3	RCT	Astra Tech OsseoSpeed	45	0	−0.04 ± 0.17	0.07 ± 0.23
	Friberg and Ahmadzai (2019)	2019	1	Prosp. study	Nobel Parallel CC	22	2	−0.41 ± 0.36	nr
	Heydecke et al. (2019)	2019	3	Prosp. study	Nobel Replace CC	90	0	−0.29 ± 0.17	nr
	Hosseini et al. (2020)	2019	5	Prosp. study	Astra Tech EV	33	0	−0.02 ± 0.07	0.07 ± 0.11
	Meijndert et al. (2020)	2019	5	Prosp. study	Straumann Bone Level implant	50	0	−0.03 ± 0.13	nr
	Zuiderveld et al. (2019)	2019	1	Prosp. study	Nobel Replace CC	40	0	0.05 ± 0.44	−0.04 ± 0.28
	Wittneben et al. (2020)	2020	3	RCT	Straumann Bone Level implant	39	0	−0.10 ± 0.17	nr
PS‐parallel	Canullo et al. (2013)	2013	2	RCT	Sweden&Martina Premium SP	20	0	−0.17 ± 0.20	nr
	Canullo et al. (2016)	2016	5	RCT	Sweden&Martina Premium SP	30	0	−0.09 ± 0.07	0.08 ± 0.11
	Canullo et al. (2018)	2018	5	Prosp. study	Sweden&Martina Premium SP	22	0	−0.06 ± 0.06	nr
	Cooper et al. (2019)	2019	3	RCT	Biomet 3i, NanoTite Certain Prevail	32	5	−0.35 ± 0.17	0.10 ± 0.23
	Lowy et al. (2019)	2019	1	RCT	BioHorizons Tapered Plus	20	0	−0.05 ± 0.21	0.10 ± 0.70
PM‐parallel	Raghoebar et al. (2009)	2009	1	RCT	Nobel Replace Groovy	45	0	−0.54 ± 0.13	‐0.63 ± 1.63
	Fu et al. (2014)	2014	1	RCT	Tapered Screw‐Vent	26	0	−1.28 ± 0.91	nr
	Felice et al. (2015)	2015	1	RCT	Dentsply Friadent, XiVE S plus	23	0	−0.19 ± 0.10	nr
	Meloni et al. (2015)	2015	1	RCT	Nobel Replace Tapered	30	0	−0.87 ± 0.19	nr
	Den Hartog et al. (2016)	2016	5	RCT	Nobel Replace Tapered Groovy	27	1	−0.03 ± 0.10	nr
	Guarnieri et al. (2016)	2016	3	Prosp. study	BioHorizons Tapered Internal	13	0	−0.14 ± 0.07	−0.01 ± 0.04
	Hsu et al. (2016)	2016	1	RCT	Zimmer dental, Tapered Screw‐Vent	13	0	−0.74 ± 0.47	nr
	Den Hartog et al. (2017)	2017	5	RCT	Nobel Tapered Groovy	54	0	−0.25 ± 0.20	nr
	Gjelvold et al. (2017)	2017	1	RCT	BioHorizons Tapered Internal	50	2	−0.63 ± 0.54	0.21 ± 0.46
	Cooper et al. (2019)	2019	3	RCT	Nobel Speedy Replace	34	5	−0.34 ± 0.17	nr
	Lowy et al. (2019)	2019	1	RCT	BioHorizons Internal	20	0	−0.65 ± 0.59	0.50 ± 0.90
	Gjelvold et al. (2020)	2020	1	Prosp. study	BioHorizons Tapered Internal	21	2	−0.4 ± 0.41	−0.02 ± 0.36

### 3.3 Risk of bias

ROBINS‐1 was used on the prospective non‐randomized trials and the domain with a high risk of bias was “bias due to confounding.” Low risk of bias was detected in “bias due to deviations from intended interventions” and in “bias in selection of the reported result.” Five studies had a high risk of bias on at least one domain (20.8%), 15 studies had a moderate risk of bias on at least one domain (62.5%), and 4 studies had a low risk of bias (16.7%) (see Figure S1).

RoB‐2.0 was applied to the RCTs and a high risk of bias was seen in the domain “bias due to deviations from intended interventions.” Low risk of bias was detected in the domain “bias due to missing outcome data.” Eighteen studies had a high risk of bias on at least 1 domain (90%), and the remaining two studies had low risk of bias (10%; see Figure S2).

### 3.4 Publication bias

The funnel plot showed no studies on the lower right part of the plot, indicating a possibility of publication bias (see Figure S3).

### 3.5 Quantitative synthesis

#### 3.5.1 Bone level change

All the study groups reported bone level changes (*n* = 48). The pooled bone level change per year for all the groups was −0.24 mm (95% CI: −0.27 to −0.20). In the PS‐conical group, it was −0.16 mm (95% CI: −0.19 to −0.13), while in the PS‐parallel group it was −0.14 mm (95% CI: −0.3 to −0.06) and in the PM‐parallel group it was −0.48 mm (95% CI: −0.63 to −0.32).

**FIGURE 2:**
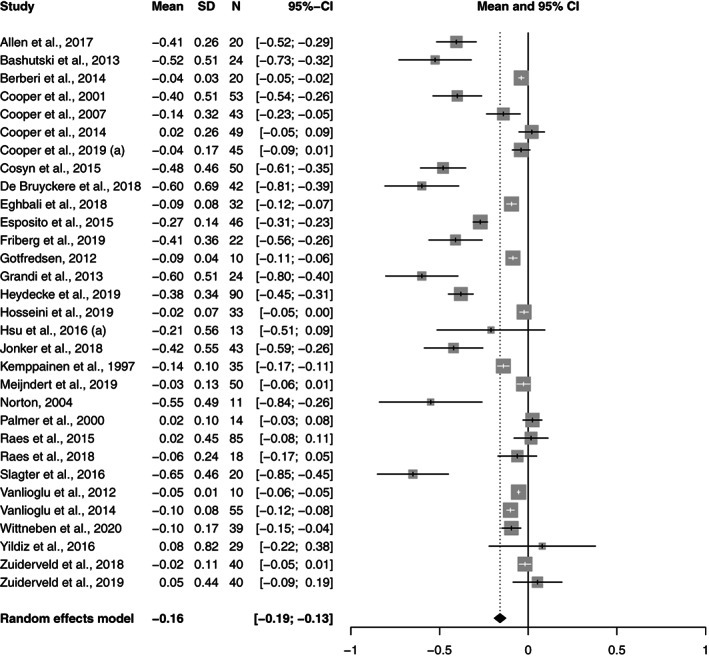
Forest plots for random effects meta‐analysis of studies evaluating bone level change in the PS‐conical group

**FIGURE 3:**
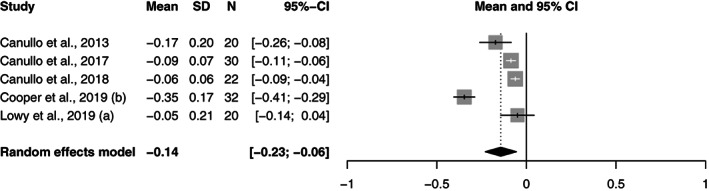
Forest plots for random effects meta‐analysis of studies evaluating of bone level change in the PS‐parallel group

**FIGURE 4:**
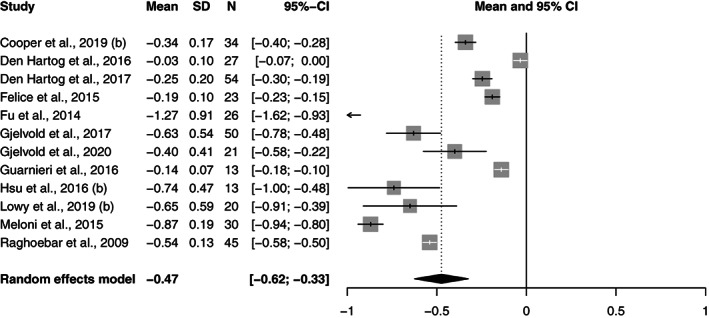
Forest plots for random effects meta‐analysis of studies evaluating of bone level change in the PM‐parallel group

A meta‐regression analysis revealed that the differences in bone change per year were statistically significant between the two platform switched and the platform matched group; PS‐conical versus PM‐parallel (*p* = 0.00), PS‐parallel versus PM‐parallel (*p* = 0.00). The difference between the conical and parallel platform switched groups (*p* = 0.52) was not significant. The Forest plots of the random effects meta‐analysis are depicted in Figures 2, 3, 4.

#### 3.5.2 Implant loss

Forty‐seven study groups reported on implant survival. The percentage of pooled implant loss per year for all the groups was 0.19 (95% CI: 0.16–0.21) with 0.13 (95% CI: 0.10–0.16) in the PS‐conical group, 0.22 (95% CI: 0.12–0.33) in the PS‐parallel group, and 0.73 (95% CI: 0.55–0.91) in the PM‐parallel group.

A meta‐regression analysis revealed a significant statistical difference in implant loss between the PS‐conical and the PM‐parallel group. There was no statistical difference found when PS‐conical versus PS‐parallel (*p* = 0.79) and PS‐parallel versus PM‐parallel (*p* = 0.10) were analyzed. Forest plots of the random effects meta‐analysis are depicted in Figure S4.

#### 3.5.3 Mid‐buccal mucosa level changes

Twenty study groups reported changes in mid‐buccal mucosa level, namely, 12 in the PS‐conical group, 3 in the PS‐parallel group, and 5 in the PM‐parallel connection group. The groups' pooled mid‐buccal mucosa level change per year was 0.05 mm (95% CI: 0.01 to 0.08), composed of 0.04 mm (95% CI: −0.02 to 0.09) in the PS‐conical group, 0.08 mm (95% CI: 0.05 to 0.12) in the PS‐parallel group, and 0.04 mm (95% CI: −0.08 to 0.16) in the PM‐parallel group. A meta‐regression analysis revealed no statistically significant difference between the three groups. Forest plots of the random effects meta‐analysis are depicted in Figure S5.

## 4 DISCUSSION

There is a relationship between the type of implant‐abutment connection configuration and peri‐implant bone loss around implants placed in the anterior region of the maxilla. The PS‐conical and PS‐parallel connection configurations are comparable with less peri‐implant bone loss than PM‐parallel connections. The results of this systematic review reveal that the presence of a platform switch seems to be of more influence on peri‐implant bone loss than the internal connection configuration itself.

In the present review, PS‐conical and PS‐parallel connection configurations result in significantly less peri‐implant bone loss than the PM‐parallel connections. However, it must be noted that this is based on three PS‐parallel groups and five PM‐parallel groups, compared to 12 PS‐conical groups. This uneven distribution might affect the statistical power of the meta‐analysis. Although the Caricasulo et al. (2018), Vetromilla et al. (2019) and Yu et al. (2020) studies confirmed that the least amount of bone loss occurs with conical connections, only Caricasulo et al. (2018) researched the effect of platform switching. They found 0.30 mm more bone loss on applying PM connections than the PS‐conical connection (significant), and that the PS‐parallel only resulted in 0.05 mm more bone loss than the conical connection (not significant). This is in agreement with our study's outcome where there is no significant difference between the PS connection, but the difference with the PM is significant. Both Vetromilla et al. (2019) and Yu et al. (2020) did not look into the effect of platform switching which might explain why Yu et al. (2020) found a significant difference between the conical and the non‐conical group and why Vetromilla et al. (2019) found fairly high amounts of bone loss in the internal‐connection group (0.7 mm). This is closer to the 0.52 mm bone loss in our PM groups than to the 0.14 mm bone loss in our PS groups. Hsu et al. (2017) did compare platform‐switching with platform‐matching. They concluded that platform switched connections are accompanied with less peri‐implant bone loss than the platform matched connections. Comparing the results of previous authors (Caricasulo et al., 2018; Hsu et al., 2017; Vetromilla et al., 2019; Yu et al., 2020) with the present review reveals that platform‐switching plays an important protective role in preserving the level of peri‐implant bone. Perhaps, supported by the Caricasulo et al. (2018) results, it can even be cautiously stated that the presence of a platform switch has more influence on bone level change than the presence or absence of a conical component in the connection.

The meta‐regression analysis of the present review revealed a significant statistical difference in implant loss between the PS‐conical and the PM‐parallel group. There was no statistical difference found when PS‐conical versus PS‐parallel (*p* = 0.79) and PS‐parallel versus PM‐parallel (*p* = 0.10) were analyzed. Hsu et al. (2017) did compare platform‐switching with platform‐matching, but they did not find a significant difference in implant survival rate. Vetromilla et al. (2019) concluded that a conical connection resulted in a higher implant survival rate than a platform‐matched configuration, which is in line with the meta‐analysis in our study.

According to our study, in contrast to bone level change platform switching seems to have little influence on the mid‐buccal mucosa level. When searching for studies that compared implant abutment connections in the aesthetic region, only one RCT (Cooper et al., 2019) was set up in the anterior maxilla and reported no statistically significant differences between the three connection types concerning mid‐buccal mucosa changes. The overall mid‐buccal retractions in both the PS‐conical, PS‐parallel and PM‐parallel groups were small and the differences were negligible.

Although there are statistically significant differences between the abutment connection configurations, all three types' clinical and radiographic results are good. All the reported bone loss, implant loss and mid‐buccal mucosa level change results are within the range of what is deemed acceptable. However, long term stability is important, especially the durability of an aesthetically good result, hence the authors favored the internal connections with a platform switch over the connections with a platform match. Yet, the included studies only had a relatively short follow‐up (mostly 1 year) and since the tissues around implants change continuously, albeit only a little, it would be useful to re‐evaluate the previous statement when more long‐term studies are available.

## 5 STRENGTH AND LIMITATIONS

The strength of this meta‐analysis is the broad and detailed literature search in multiple databases. A limitation to this study is that the quality of the reporting in the included studies was weak and the median follow‐up time was short (1 [1;5] year). Also, the meta‐analysis was done for variables that can be measured in many ways (in particular bone level change and mucosa level change) and are subject to confounding factors (such as surgical and restauration protocol and implant geometry) and was thus subject to heterogeneity, which means that the outcome must be viewed with caution. Another limitation is the decision to calculate annual bone loss, annual implant loss and annual mucosa change rates which, although good for comparability purposes, resembles a linear relation that assumes that the same quantity of bone, implants or mucosa is lost every year. Yet, in real life, most remodeling takes place in the first year, and only a few changes in the years thereafter. We accepted this limitation in order to perform a meta‐analysis and this approach is commonly accepted in the dental implant literature, but one should still interpret the results with caution.

## 6 RECOMMENDATIONS FOR FUTURE RESEARCH

Due to a lack of well‐designed RCTs and high quality studies, additional well designed studies are needed to be able to truly rate the effect of different implant‐abutment connections in the aesthetic zone. We therefore encourage efforts to come to a consensus on how to measure and report clinical and radiographic variables, as well as aesthetic ratings, accurately and homogenously.

## 7 CONCLUSION

The performance of conical and parallel connection configurations with a platform switch is comparable regarding peri‐implant bone loss and implant loss when applied to solitary implant restorations in the aesthetic zone. Parallel walled platform matched connections showed the most bone level change and implant loss. None of the connection configurations was significantly better at preserving the mid‐buccal mucosa levels.

## CONFLICT OF INTEREST

The authors declare to have no conflict of interest with the contents of this article.

## AUTHOR CONTRIBUTIONS

Caroliene M. Meijndert: concept/design, execution of search and data analysis/interpretations, drafting of article, final approval, accountable for all aspects of the work. Gerry M. Raghoebar: critical revision of article, final approval. Arjan Vissink: critical revision of article, final approval. Konstantina Delli: concept/design, data analysis/interpretation, drafting of article, critical revision of article, final approval, accountable for all aspects of the work. Henny J. A. Meijer: data collection, concept/design, statistics, data analysis/interpretation, drafting of article, final approval, accountable for all aspects of the work.

## Supporting information


**Figure S1** Visualization risk‐of‐bias assessments ROBINS‐1 for prospective non‐randomized trials
**Figure S2**: Visualization risk‐of‐bias assessment RoB‐2 for randomized controlled trials
**Figure S3**: Funnel plot of standard error by log odds ratio.
**Figure S4**: Forest plots for random effects meta‐analysis of studies evaluating implant loss in the PS‐Conical group
‐ Forest plots for random effects meta‐analysis of studies evaluating implant loss in the PS‐parallel group
‐ Forest plots for random effects meta‐analysis of studies evaluating implant loss in the PM‐parallel group
**Figure S5**: Forest plots for random effects meta‐analysis of studies evaluating mid‐buccal mucosa level change in the PS‐Conical group.
‐ Forest plots for random effects meta‐analysis of studies evaluating mid‐buccal mucosa level change in the PS‐parallel group
‐ Forest plots for random effects meta‐analysis of studies evaluating mid‐buccal mucosa level change in the PM‐parallel group

## Data Availability

The data that support the findings of this study are available from the corresponding author upon reasonable request.
