# Supplementary material for: The effect of implant‐abutment connections on peri‐implant bone levels around single implants in the aesthetic zone: A systematic review and a meta‐analysis
Source: Clin Exp Dent Res. 2021 Aug 21;7(6):1025–36. doi: 10.1002/cre2.471 (PMC8638280; doi:10.1002/cre2.471)

Study	Mean	SD	N	95%−CI
Cooper et al., 2001	0.34	0.94	53	[0.09; 0.59]
Cooper et al., 2007	0.17	0.47	43	[0.03; 0.31]
Cooper et al., 2014	0.08	0.21	49	[0.02; 0.14]
Cooper et al., 2019 (a)	0.07	0.23	45	[0.00; 0.13]
Cosyn et al., 2015	0.06	0.60	50	[−0.11; 0.23]
De Bruyckere et al., 2018	−0.17	0.33	42	[−0.28; −0.07]
Eghbali et al., 2018	−0.02	0.07	32	[−0.05; 0.00]
Hosseini et al., 2019	0.07	0.11	33	[0.03; 0.10]
Raes et al., 2015	0.10	0.45	85	[0.00; 0.19]
Zuiderveld et al., 2018	−0.10	1.19	40	[−0.47; 0.26]
Zuiderveld et al., 2019	−0.04	0.28	40	[−0.13; 0.05]
**Random effects model**	**0.04**			**[−0.02; 0.09]**

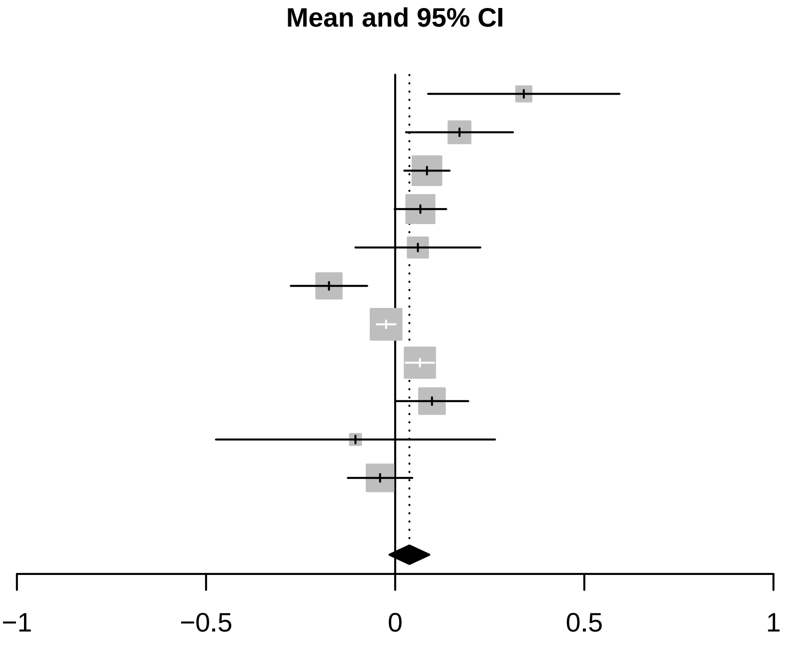

Supplement: Supplementary file 1 — Figure S1 Visualization risk‐of‐bias assessments ROBINS‐1 for prospective non‐randomized trials Figure S2: Visualization risk‐of‐bias assessment RoB‐2 for randomized controlled trials Figure S3: Funnel plot of standard error by log odds ratio. Figure S4: Forest plots for random effects meta‐analysis of studies evaluating implant loss in the PS‐Conical group ‐ Forest plots for random effects meta‐analysis of studies evaluating implant loss in the PS‐parallel group ‐ Forest plots for random effects meta‐analysis of studies evaluating implant loss in the PM‐parallel group Figure S5: Forest plots for random effects meta‐analysis of studies evaluating mid‐buccal mucosa level change in the PS‐Conical group. ‐ Forest plots for random effects meta‐analysis of studies evaluating mid‐buccal mucosa level change in the PS‐parallel group ‐ Forest plots for random effects meta‐analysis of studies evaluating mid‐buccal mucosa level change in the PM‐parallel group [file CRE2-7-1025-s001.zip › CRE2_471_CRE2_471_cre2.20210147-File012.pdf]
