# Supplementary material for: The effect of implant‐abutment connections on peri‐implant bone levels around single implants in the aesthetic zone: A systematic review and a meta‐analysis
Source: Clin Exp Dent Res. 2021 Aug 21;7(6):1025–36. doi: 10.1002/cre2.471 (PMC8638280; doi:10.1002/cre2.471)

Study	Mean	SD	N	95%−CI
Cooper et al., 2019 (b)	0.00	0.33	34	[−0.11; 0.11]
Gjelvold et al., 2017	0.21	0.46	50	[0.08; 0.34]
Gjelvold et al., 2020	−0.02	0.36	21	[−0.17; 0.13]
Guarnieri et al., 2016	−0.01	0.04	13	[−0.03; 0.01]
Lowy et al., 2019 (b)	0.50	0.90	20	[0.11; 0.89]
Raghoebar et al., 2009	−0.63	1.63	45	[−1.11; −0.15]
**Random effects model**	**0.04**			**[−0.08; 0.16]**

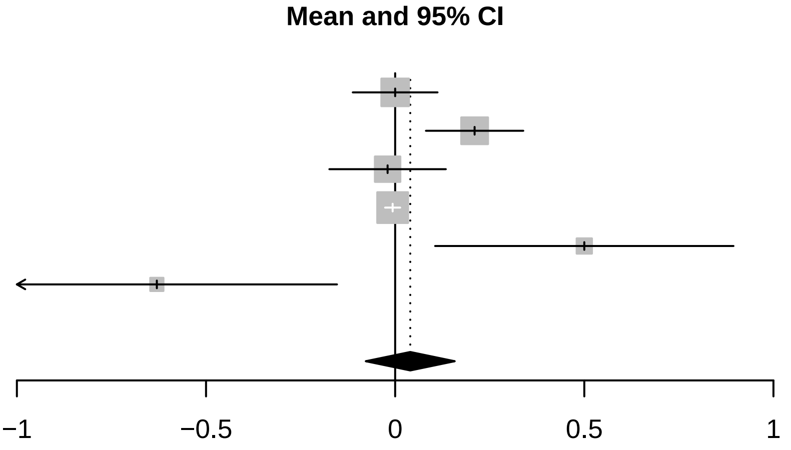

Supplement: Supplementary file 1 — Figure S1 Visualization risk‐of‐bias assessments ROBINS‐1 for prospective non‐randomized trials Figure S2: Visualization risk‐of‐bias assessment RoB‐2 for randomized controlled trials Figure S3: Funnel plot of standard error by log odds ratio. Figure S4: Forest plots for random effects meta‐analysis of studies evaluating implant loss in the PS‐Conical group ‐ Forest plots for random effects meta‐analysis of studies evaluating implant loss in the PS‐parallel group ‐ Forest plots for random effects meta‐analysis of studies evaluating implant loss in the PM‐parallel group Figure S5: Forest plots for random effects meta‐analysis of studies evaluating mid‐buccal mucosa level change in the PS‐Conical group. ‐ Forest plots for random effects meta‐analysis of studies evaluating mid‐buccal mucosa level change in the PS‐parallel group ‐ Forest plots for random effects meta‐analysis of studies evaluating mid‐buccal mucosa level change in the PM‐parallel group [file CRE2-7-1025-s001.zip › CRE2_471_CRE2_471_cre2.20210147-File014.pdf]
