# Supplementary figures and images for: The effect of implant‐abutment connections on peri‐implant bone levels around single implants in the aesthetic zone: A systematic review and a meta‐analysis
Source: Clin Exp Dent Res. 2021 Aug 21;7(6):1025–36. doi: 10.1002/cre2.471 (PMC8638280; doi:10.1002/cre2.471)

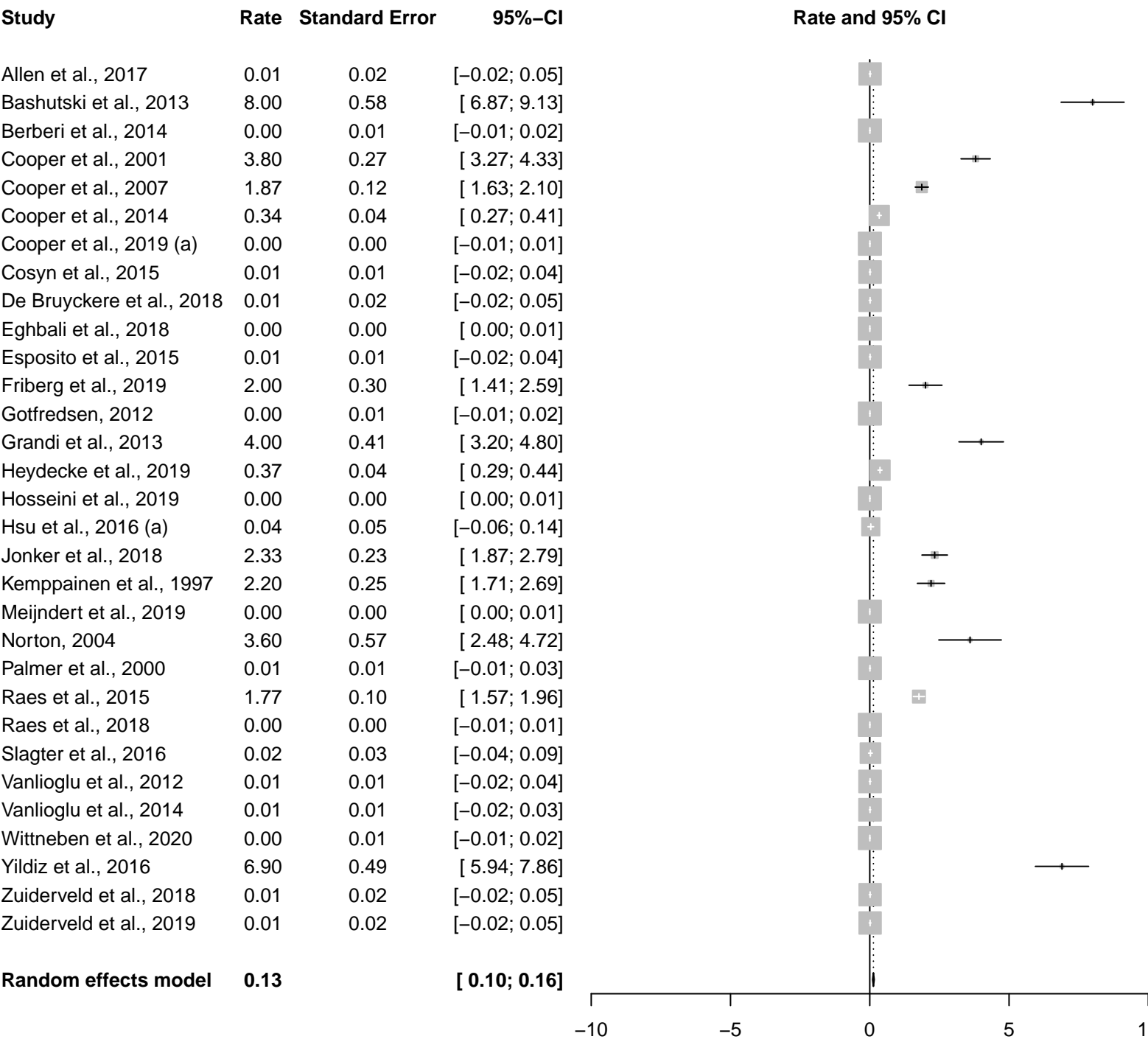

Supplement: Supplementary file 1 — Figure S1 Visualization risk‐of‐bias assessments ROBINS‐1 for prospective non‐randomized trials Figure S2: Visualization risk‐of‐bias assessment RoB‐2 for randomized controlled trials Figure S3: Funnel plot of standard error by log odds ratio. Figure S4: Forest plots for random effects meta‐analysis of studies evaluating implant loss in the PS‐Conical group ‐ Forest plots for random effects meta‐analysis of studies evaluating implant loss in the PS‐parallel group ‐ Forest plots for random effects meta‐analysis of studies evaluating implant loss in the PM‐parallel group Figure S5: Forest plots for random effects meta‐analysis of studies evaluating mid‐buccal mucosa level change in the PS‐Conical group. ‐ Forest plots for random effects meta‐analysis of studies evaluating mid‐buccal mucosa level change in the PS‐parallel group ‐ Forest plots for random effects meta‐analysis of studies evaluating mid‐buccal mucosa level change in the PM‐parallel group [file CRE2-7-1025-s001.zip › CRE2_471_CRE2_471_cre2.20210147-File009.pdf]

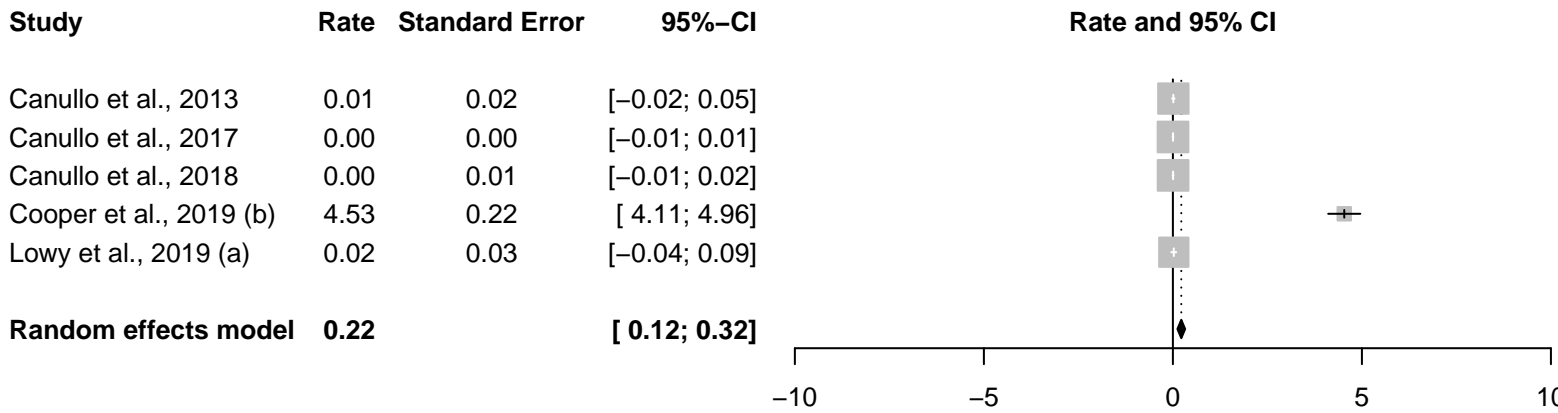

Supplement: Supplementary file 1 — Figure S1 Visualization risk‐of‐bias assessments ROBINS‐1 for prospective non‐randomized trials Figure S2: Visualization risk‐of‐bias assessment RoB‐2 for randomized controlled trials Figure S3: Funnel plot of standard error by log odds ratio. Figure S4: Forest plots for random effects meta‐analysis of studies evaluating implant loss in the PS‐Conical group ‐ Forest plots for random effects meta‐analysis of studies evaluating implant loss in the PS‐parallel group ‐ Forest plots for random effects meta‐analysis of studies evaluating implant loss in the PM‐parallel group Figure S5: Forest plots for random effects meta‐analysis of studies evaluating mid‐buccal mucosa level change in the PS‐Conical group. ‐ Forest plots for random effects meta‐analysis of studies evaluating mid‐buccal mucosa level change in the PS‐parallel group ‐ Forest plots for random effects meta‐analysis of studies evaluating mid‐buccal mucosa level change in the PM‐parallel group [file CRE2-7-1025-s001.zip › CRE2_471_CRE2_471_cre2.20210147-File010.pdf]

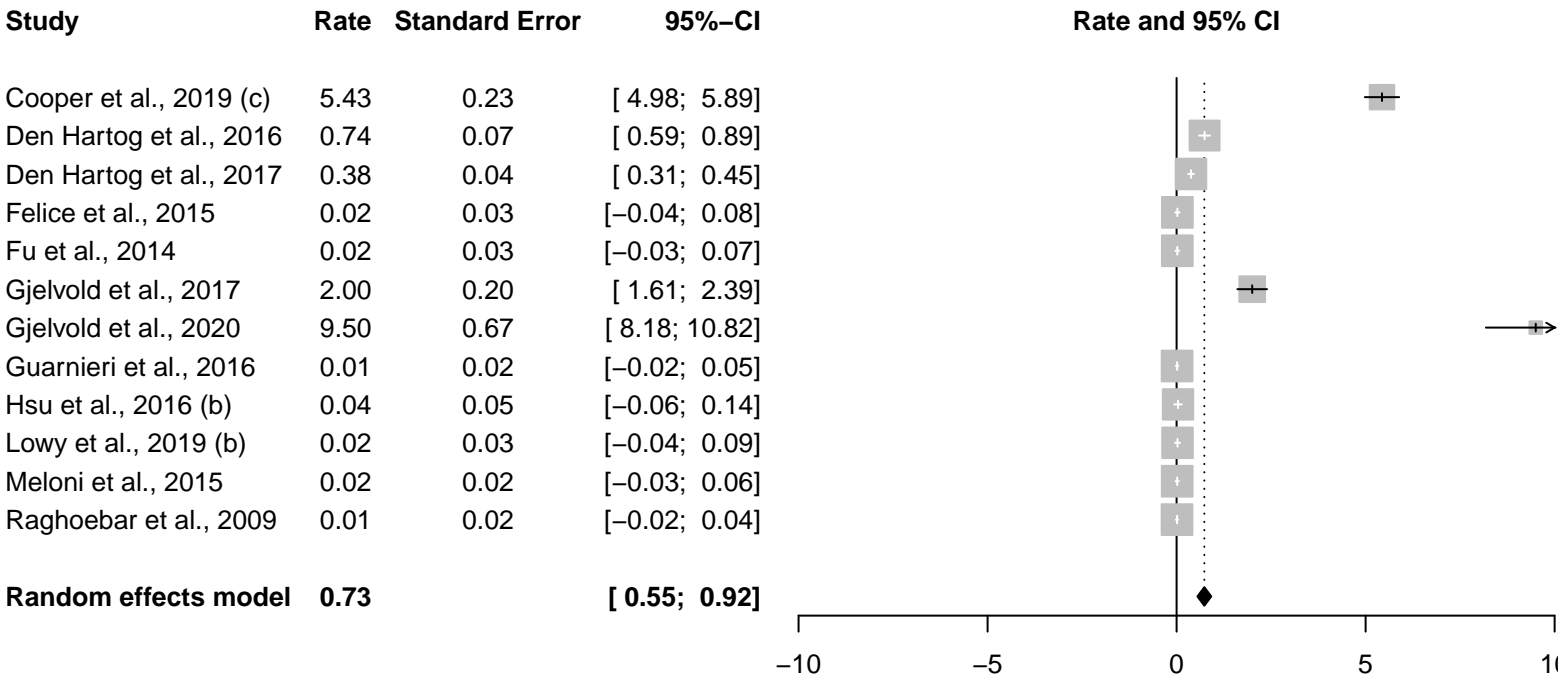

Supplement: Supplementary file 1 — Figure S1 Visualization risk‐of‐bias assessments ROBINS‐1 for prospective non‐randomized trials Figure S2: Visualization risk‐of‐bias assessment RoB‐2 for randomized controlled trials Figure S3: Funnel plot of standard error by log odds ratio. Figure S4: Forest plots for random effects meta‐analysis of studies evaluating implant loss in the PS‐Conical group ‐ Forest plots for random effects meta‐analysis of studies evaluating implant loss in the PS‐parallel group ‐ Forest plots for random effects meta‐analysis of studies evaluating implant loss in the PM‐parallel group Figure S5: Forest plots for random effects meta‐analysis of studies evaluating mid‐buccal mucosa level change in the PS‐Conical group. ‐ Forest plots for random effects meta‐analysis of studies evaluating mid‐buccal mucosa level change in the PS‐parallel group ‐ Forest plots for random effects meta‐analysis of studies evaluating mid‐buccal mucosa level change in the PM‐parallel group [file CRE2-7-1025-s001.zip › CRE2_471_CRE2_471_cre2.20210147-File011.pdf]

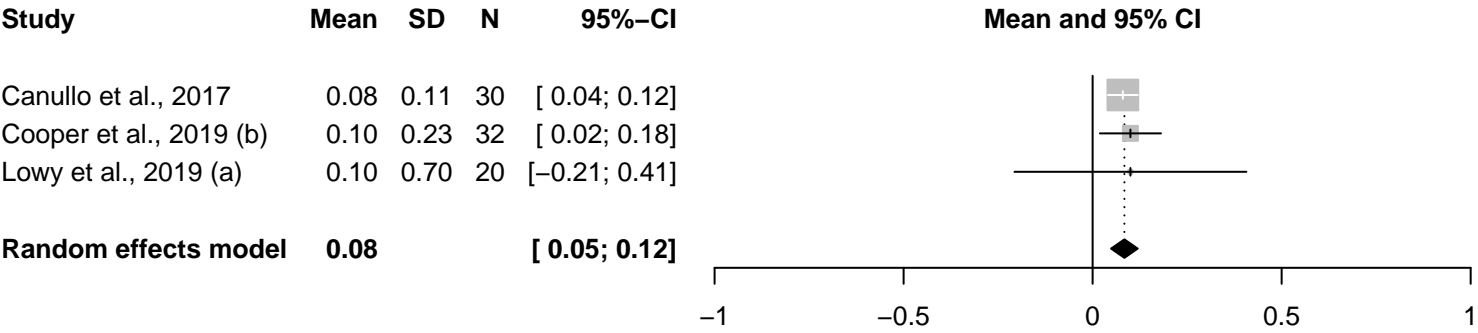

Supplement: Supplementary file 1 — Figure S1 Visualization risk‐of‐bias assessments ROBINS‐1 for prospective non‐randomized trials Figure S2: Visualization risk‐of‐bias assessment RoB‐2 for randomized controlled trials Figure S3: Funnel plot of standard error by log odds ratio. Figure S4: Forest plots for random effects meta‐analysis of studies evaluating implant loss in the PS‐Conical group ‐ Forest plots for random effects meta‐analysis of studies evaluating implant loss in the PS‐parallel group ‐ Forest plots for random effects meta‐analysis of studies evaluating implant loss in the PM‐parallel group Figure S5: Forest plots for random effects meta‐analysis of studies evaluating mid‐buccal mucosa level change in the PS‐Conical group. ‐ Forest plots for random effects meta‐analysis of studies evaluating mid‐buccal mucosa level change in the PS‐parallel group ‐ Forest plots for random effects meta‐analysis of studies evaluating mid‐buccal mucosa level change in the PM‐parallel group [file CRE2-7-1025-s001.zip › CRE2_471_CRE2_471_cre2.20210147-File013.pdf]
